# The Influence of Drying Methods on the Chemical Composition and Body Color of Yellow Mealworm (*Tenebrio molitor* L.)

**DOI:** 10.3390/insects12040333

**Published:** 2021-04-08

**Authors:** Letlhogonolo Selaledi, Monnye Mabelebele

**Affiliations:** 1 Department of Agriculture and Animal Health, College of Agriculture and Environmental Science, University of South Africa, Florida Campus, 28 Pioneer Ave, Florida Park, Roodepoort 1709, South Africa; letlhogonolo.selaledi@up.ac.za; 2 Department of Zoology and Entomology, Mammal Research Institute, Faculty of Natural and Agricultural Sciences, University of Pretoria, Hatfield, Pretoria 0002, South Africa

**Keywords:** sun-drying, nutrient, freeze-drying, amino acids, edible insects

## Abstract

**Simple Summary:**

Edible insects are rich in nutrients and moisture and this may cause high microbial growth. Insects such as mealworms must be dried to preserve their quality; different drying methods have been applied before. The energy cost of drying mealworms varies according to the drying technique that is used. Thus, the study sought to investigate the different drying procedures and their impact on the chemical composition and body color of yellow mealworm larvae. The yellow mealworm samples were gently frozen in a −20 freezer afterwards blanched and exposed to sun-drying, oven-drying, and freeze-drying then were later analyzed for their chemical composition. The crude protein content of freeze and oven-dried mealworms were similar; however, higher than those of the sun-dried samples. The color of the sun-dried mealworms changed slightly to brownish this could probably be related to Maillard reaction. The majority of the essential amino acids were higher in the sun-dried mealworms than both the oven and freeze-dried samples. It can be concluded that sun drying had the same nutritional composition as freeze and oven drying despite the color changes. Oven and freeze-drying strategies can be used to formulate mealworm-based feed and food products without noticeable nutritional changes. However, it is important to monitor and determine the microbial growth so the final product whether it meets the food safety standard.

**Abstract:**

To preserve the quality of the yellow mealworm, different drying methods are being explored by farmers and processors. However, the energy costs associated with these methods are usually high for smallholder insect-rearing farmers. Thus, the core aim of this study was to investigate different drying procedures and their impact on the chemical composition of yellow mealworm larvae. Yellow mealworms (exposed to sun, oven and freeze drying) were later analyzed for their chemical composition and body color. Crude protein (CP) content of freeze and oven-dried mealworms were similar (*p* > 0.05), but higher (*p* < 0.05) than those of the sun-dried samples. The b (yellowness) color of the sun-dried samples scored the lowest value (*p* < 0.05) in comparison with both oven and freeze-dried samples. The majority of the essential amino acids were higher (*p* < 0.05) in the sun-dried mealworms than both oven and freeze-dried samples. Similarly, the fat content of sun-dried mealworms was higher (*p* < 0.05) than if they had been oven or freeze dried. However, SFA (saturated fatty acids), PUFA (polyunsaturated fatty acids) and *n*-6 fatty acids were similar (*p* > 0.05) for all drying methods. We, therefore, conclude that sun drying resulted in the same nutritional composition as freeze and oven drying despite the noted color changes. Freeze and oven-drying strategies can be used to formulate mealworm-based feed and food products without noticeable nutritional changes. For the benefit of small-scale insect-rearing farmers, an appropriate drying technology that is affordable and easy to use should be developed considering the needs and experiences of these farmers.

## 1. Introduction

The world population is expected to gradually increase, this will have an impact on food security and therefore, lead to increasing demand for animal protein [1]. This presents an urgent need to increase the supply of sustainably sourced protein. To meet the needs and goals of the society, different methods of producing food and feeds must be proposed following the guidelines of the sustainable development goals [2]. However, the food and feed produced should also adhere to the local manufacturing standards. In the South African context, animal feeds should be produced in accordance with Fertilizers, Farm Feeds, Agricultural Remedies and Stock Remedies Act no. 36 of 1947 [3]. According to Niassy, Ekesi, Hendriks and Haller-Barker [4], South Africa has legal tools to address the use of insects as feed and food; however, there is a need to outline specifically the law in relation to the inclusion of edible insects. Recently, the growing interest of including insects in animal feeds and human food has been receiving much attention [5]. It was suggested as early as 1975 by Meyer-Rochow that insects could help to ease the problem of global food shortages and that such an approach ought to be supported by international organizations such as World Health Organization and Food and Agriculture Organization of the United Nations [6]. Several European, American, Asian and African countries are using insects as food and feed [5,7,8,9,10]. In South Africa, some companies have also started rearing insects for the animal feed industry [11]. Therefore, a sustainable industrial production technology that is efficient in terms of manufacturing standards needs to be implemented, which will produce the desired final product [12].

Insects such as mealworms must be dried to preserve their quality and other researchers have before applied different drying methods [13,14,15]. In most cases, the larvae are gently killed by freezing followed by blanching which is normally applied to preserve and store larvae [16,17]. The drying method needs to be implemented because yellow mealworms provide a favorable environment for microbial growth mainly because of the moisture and nutritive status. This can also cause Maillard reaction and lead to product degradation [16,18]. However, insects are associated with high microbial growth of commensal, pathogenic and spoilage fungi that might result from unhygienic processing methods [19]. Therefore, it is important to determine the microbial quality of dried yellow mealworms. Ramashia, Tangulani, Mashau and Nethathe [19] detected low levels of coliforms, *Salmonella, Staphylococcus aureus, Escherichia coli* and fungi in South Africa at a local dried insects market. Even though the microbial counts were within health and food standard specifications, their occurrence is still worrying.

It has been reported that yellow mealworms contain a high level of mono and polyunsaturated fatty acids as well as lipids and protein [20,21,22]. Apart from nutritional benefits, they also produce lower emission of greenhouse gases and ammonia as compared with traditional livestock [23]. Traditional insect drying methods such as sun drying have been practiced for ages. In Southern Africa, the mopane worm (*Gonimbrasia belina*) normally undergoes a fasting period that is followed by blanching and a few days of sun drying before being consumed. However, it is believed that insect contamination that occurs during the sun-drying process is because of poor storage, microbiota, and contact with the soil [17]. Therefore, blanching is normally recommended because it reduces the bacterial load [24]. As insects are being introduced to animal feeds and human food, several methods of processing are being explored. Nyangena et al. [25] studied the effects of traditional processing techniques such as boiling, toasting, oven-drying and solar-drying of four edible insect species used for food and feed in East Africa. The insects studied are adult house crickets (*Acheta domesticus*), black soldier fly (*Hermetia illucens*), African cotton leafworm (*Spodoptera littoralis*) and adult grasshoppers (*Ruspolia differens*) [25]. Furthermore, Kroncke at al. [18] studied effect of different drying methods on nutrient quality of yellow mealworm; however, the study did not include the sun drying as part of the drying method. Sun-drying as a traditional processing technique has been applied on most edible insects including termites, grasshoppers, mopane, locusts and crickets [26]. Processing techniques such as microwave drying, freeze drying, oven drying, vacuum drying and rack oven drying have been applied on yellow mealworms before [14,15,18]. The nutritional composition of yellow mealworms has been studied, numerous studies have documented their proximate analysis, amino acids and fatty acids [27,28,29,30]. However, information related to sun drying of yellow mealworms is limited or no case can be found in the scientific research. There are several technological types of equipment such as those used for oven and freeze drying that are currently being used to dry and preserve the quality of the insects. The energy cost of drying the mealworm varies according to the drying technique used. A study conducted by Kroncke, Böschen, Woyzichovski, Demtröder and Benning [18] found that the cost of oven drying is approximately R59.12/kg (€3.24), followed by freeze-drying at R52.55/kg (€2.88) and R12.23/kg (€0.67) for rack-oven drying.

To enable long-term storage of mealworms, they are either blanched or frozen after rearing to kill them gently and, in addition, they might be freeze dried or oven dried. The operational costs of the oven and freeze-drying are high [24] and therefore alternative ways for processing insects need to be explored. Most smallholder farmers in sub-Saharan Africa are poor [31]; therefore, it might be a challenge for them to acquire such apparatus. The sun-drying method is cost-effective, easily accessible, relevant and pragmatic in the context of small-scale insect rearing. However, sun-dried, edible insects such as mopane worm, termites and stinkbug were reported to have high levels of coliforms count, *E. coli, Staphylococcus. aureus* and *Salmonella* spp. [19]. Pathogenic bacteria such as *E. coli* and *S aureus* can cause a pathological condition in animals and humans. Therefore, the issue of microbiological quality and food safety of sun-dried edible insects is another area of concern. Microbiological testing plays a key role in identifying potential threats. Vandeweyer et al. [24] investigated blanching and chilled preservation of mealworm. It was reported that blanched mealworms can be stored in chilled condition for approximately seven days without microbial growth. Therefore, it can be assumed that blanching resembles a pasteurization treatment. However, spore-forming bacteria such as *clostridium* spp. have already been encountered in yellow mealworms and certain strains can cause food poisoning in humans [24,32]. Furthermore, food color is one other factor that can determine consumer acceptability. Several studies have investigated the effect of drying methods such as oven and freeze drying on the color of yellow mealworm [18,26]. However, the food color of edible insects has been poorly analyzed [26]. Additionally, information related to the color of sun-dried yellow mealworms is limited. The different drying techniques and microbial testing of mealworms have recently been studied [18,33,34], however, information related to sun drying of mealworm and its impact on color and chemical composition is limited. The focus of the study was to compare the effect of sun, oven and freeze-drying methods on the chemical composition and color of yellow mealworm larvae.

## 2. Materials and Methods

### 2.1. Sample Acquisition and Preparation

Yellow mealworm larvae (*Tenebrio molitor* Linnaeus, 1758) used in this study were obtained from the University of Pretoria, Department of Zoology and Entomology (Pretoria, South Africa). Mealworms larva which are the larval form of the mealworm beetle, *Tenebrio molitor* L., a species of darkling beetle. The larval stage used during the experiment was between 9th and 10th instar. The mealworms were stored in an environmentally controlled chamber at 25 °C with relative moisture of 55–60% and were fed ad libitum with wheat bran and gem squash. The research was approved by the Human Research Ethics Committee of the College of Agriculture and Environmental Science at the University of South Africa with ethical clearance number: 2019/CAES_HREC/138.; Larvae of yellow mealworm were removed and individually frozen at −20 °C for 24 h; afterwards, yellow mealworms were blanched for 20 s in boiling water.

### 2.2. Drying 

The yellow mealworm larvae were processed by either sun drying, oven drying or freeze drying. For sun drying, samples of 100 g of larvae were kept in the direct sun on a ceramic plate covered with a mesh for a period of 24 h, the daily ambient air temperature recorded was between 27 and 30 °C and direct sun temperature maximum was 43 °C. The samples were later kept in polyethylene bags and stored at a room temperature of ±20 °C. The freeze dryer condenser (BenchtopPRO^®^, CRC Industries, Inc., Horsham, PA, USA) was set to −50 °C and 100 g of larvae were sectioned into stainless-steel plates (diameter: 36 cm) and a vacuum was applied for 24 h and later kept at room temperature after being separately packed in closed polyethylene bags. An EcoTherm oven was used to dry 100 g of mealworms on an oven-safe glass plate (60 × 80 × 2 cm) in the middle of the oven at 120 °C for 1 h at ventilation stage 2. After drying, the dried larvae were independently packed in secure polyethylene bags and submitted to different laboratories for analysis. The freeze-drying processes involve the freezing stage at which the sample is completely frozen, followed by the primary drying in which the water sublimates in a vacuum and lastly, the secondary drying in which the remaining liquid water is vaporized [12].

### 2.3. Proximate Analysis

Crude fiber, moisture, ash and gross energy were carried out following the standard methods of the Association of Official Analytical Chemists [35]. Crude protein (N × 6.5) content was examined by the Dumas method by means of a nitrogen analyzer [36]. A Memmert UM500 dry oven was used to determine the dry matter content by dehydrating mealworm samples at 105 °C overnight. In order to determine the ash content, the samples were incinerated. 

### 2.4. Color Analysis

To determine the color of the mealworms, a color measurements were performed on whole dried mealworm using a colorimeter (CM-5; Konica Minolta, Tokyo, Japan). A total of 10 g of freeze dried; oven dried and sun-dried mealworms was placed into small cups, and the color values were detected under D65-100. The browning indices and color difference (Δ*E*^∗^)were calculated according to the formula described by Lenaerts et al. [15].

(1) $$\;\Delta E^{*}=\;\sqrt{\Delta a^{*2}+\;\Delta b^{*2}+\Delta L^{*2}}$$

### 2.5. Amino Acid Analysis

The Waters Acquity Ultra performance liquid chromatography (UPLC) system was used to analyze the amino acid with a photodiode array (PDA) detector. The AccQ-Tag ultra-amino acid kit from Waters was used in the process of determining the amino acids and the derivatization kit which contains 5 vials was also included. Preparation of derivatising agent (AQC): 1 mL of dry acetonitrile (in a sealed vial from the kit) was added to the reagent—a vial containing 3 mg of AQC. This vial was then heated, vortexed and sonicated to ensure the reagent dissolved completely. Since 20 µL of a derivatizing agent are used for each sample, this 1 mL volume is sufficient for 50 reactions. Since the derivatizing agent is prone to hydrolysis, it should be stored in a desiccator where it is normally stable for approximately one week. Hewitson, Wheat and Diehl’s (2017) [37] method was employed to determine amino acids. Because of the strength of the acid used during hydrolysis, it is unlikely that the buffer used will be strong enough to neutralize the remaining HCl. Therefore, all hydrolyzed samples were treated with 6 M NaOH to adjust the pH. For higher protein concentrations than 7%, an initial 5× dilution of the hydrolysate was performed (200 µL sample + 200 µL NaOH + 600 µL H2O). This initial dilution was then used to prepare any subsequent dilutions.

### 2.6. Fatty Acid Composition

The Folch method [38] was used to determine the total lipid from yellow mealworm. The fat was dried using a rotary evaporator and the extracts were dried overnight in a vacuum oven at 50 °C. After extracting the fat, it was expressed as a percentage of fat (*w*/*w*) per 100 g tissue. The residue was weighed on a filter paper that is normally used for Folch extraction and the fat-free dry matter (FFDM) content was examined. The fatty acid analysis was carried out according to Park and Goins [39]. Fatty acids were measured as the quantity of each individual fatty acid to the total acids present in the sample. Omega-3 (*n*-3) fatty acids, omega-6 (*n*-6) fatty acids, total saturated fatty acids (SFA), total mono-unsaturated fatty acids (MUFA), polyunsaturated fatty acids (PUFA), PUFA/SFA ratio (P/S) and *n*-6/*n*-3 ratio were all determined.

### 2.7. Statistical Analysis 

Data collected were analyzed using a one-way analysis of variance of statistical software [40]. The difference between treatments was determined by Duncan’s Multiple Range test using a *p*-value < 0.05 as significance threshold limit. 

## 3. Results

### 3.1. Proximate Analysis

The results of the chemical composition of yellow mealworm exposed to different drying methods are presented in Table 1. The standard error of the mean (SEM) is also presented. The dry matter of the oven-dried yellow mealworm was higher (*p* < 0.05) than that of the sun and freeze-dried mealworm. However, the crude protein content of the sun-dried mealworms was lower (*p* < 0.05) compared to the freeze and oven-dried mealworms. Any of the other parameters—ash, fiber and gross energy were very similar (*p* > 0.05) despite small fluctuation ranges. 

### 3.2. Color Analysis

The color parameters of dried mealworms are shown in Table 2. The values of L (lightness) of the mealworm ranged from 30.95 to 42.02 and the freeze-dried samples’ value of L* was higher (*p* < 0.05) compared to both sun and oven-dried samples. The a* (redness) value was similar (*p* > 0.05) despite small fluctuations. The b* values (yellowness) of the sun dried had the lowest value (*p* < 0.05) compared to both oven and freeze-dried samples. The color development was probably due to the Maillard reaction, caramelization and phospholipid degradation [15,18]. 

### 3.3. Mineral Composition 

Depending on the drying strategy, the different mineral compositions were analyzed, and the results are presented in Table 3. Oven-dried mealworms exhibited higher (*p* < 0.05) calcium, magnesium, copper and sodium contents compared to those found in either sun or freeze-dried mealworms. However, sun drying exhibited higher (*p* < 0.05) manganese, zinc, sodium and phosphorus values than those of the freeze-dried mealworms. Only the iron content of mealworms exposed to freeze-drying was higher (*p* < 0.05) than that of sun and oven-dried mealworms.

### 3.4. Amino Acid Profile of Yellow Mealworm Exposed to Different Drying Methods

Table 4 depicts the amino acid values of the dried mealworm larvae. Generally, sun-dried mealworms exhibited higher (*p* < 0.05) amino acid profiles with the exception of tyrosine. Histidine values of sun and freeze-dried mealworms were similar (*p* > 0.05) but higher (*p* < 0.05) than those that were oven dried. Moreover, glycine, alanine and methionine contents of sun and oven-dried mealworms were similar (*p* > 0.05) but higher (*p* < 0.05) than those that were freeze dried. However, lysine, valine, isoleucine and threonine contents of sun-dried mealworm samples were higher (*p* < 0.05) compared to both freeze and oven-dried samples. The content of tryptophan was not determined in this study. 

### 3.5. Fatty Acid Composition

The fatty acid composition of the different dried mealworm samples examined is shown in Table 5. Similar values (*p* > 0.05) were obtained for almost all fatty acids irrespective of the drying strategy applied. Inclusively, oleic, palmitic and linoleic acids were the major fatty acid components in quantity. The total saturated fatty acids and total omega 6 fatty acid (*n*-6) had similar (*p* > 0.05) values irrespective of the drying method used. The moisture content of the fatty acid analysis was higher (*p* < 0.05) in the freeze-dried sample (21.84) as compared to oven dried (6.35) and sun-dried (3.39) samples. Based on the quantitative and qualitative analysis the fat percentage of sun-dried samples was higher (*p* < 0.05) compared to both freeze and oven-dried samples. The SFA values of mealworms exposed to different drying strategies were similar (*p* > 0.05) regardless of small variations. The MUFA contents of oven-dried samples were significantly higher (*p* < 0.05) compared to sun and freeze-dried mealworms. The total omega 6 fatty acids content was also similar (*p* > 0.05) amongst the different drying methods despite marginal differences.

## 4. Discussion

Mealworm drying is a process that is used to slow down or inhibit microbial growth, browning reaction and enzymatic activity [24]. Therefore, this study investigated the influence of different drying methods on the chemical composition and color of yellow mealworms. The crude protein and ash of yellow mealworm irrespective of the drying methods observed in this study were within the ranges found by other research reports [14,15,41]. Although the protein and ash were slightly higher than those obtained by Jones, Cooper and Harding [42] and Siemianowska et al. [10]. Different processing methods and the larval stage utilized for the analysis could influence the results [43]. There are many other factors such as rearing conditions and diet composition that can influence the chemical and nutritional quality of mealworms [44,45]. The comparison between the protein content of freeze or oven-dried mealworm samples have been documented however, the protein content of sun-dried mealworm is limited in the literature. In this study, it was found that the crude protein of sun-dried mealworms were lower compared to both freeze and oven-dried samples. This could have been influenced by the dry matter base of the samples. Similar results of the lower protein content of sun-dried insect meals were reported by other researchers [43,44,45,46]. According to Son, Lee, Hwang, Nho and Kim [47], the fat content of the mealworm may have a significant influence on the color measurement. The change in b value was probably due to browning substances produced during the sun drying method [14,18]. Other heating methods, such as oven drying, have been reported to cause an increase in color concentration as a result of phospholipid deprivation [48].

Minerals are inorganic nutrients required in smaller quantities, depending on the species. The mineral composition of dried mealworms was within the ranges found in a study by Elahi et al. [28]. The different reported values for the mineral composition of dried mealworms in literature can be described by changes in feed substrate, rearing circumstances or various systems of the analysis used [49]. The calcium values obtained in the current study were below the recommended daily intake for humans and poultry. Food containing calcium levels above 240 mg/100 g are considered well enough for human consumption [50]. The results of this study agreed with those performed by Jajic et al. [51] with regard to the calcium content of the mealworm meal. However, in the present study, the phosphorus and magnesium content were higher (6.8% and 2.2%, respectively) compared to the findings of Jajic et al. [51]. The recommended daily intake of magnesium for an adult is within the ranges of 220–260 mg, therefore, consuming about 200 g of mealworm in a day will surpass the daily requirements. Furthermore, zinc, iron and potassium in the current study, were within the ranges reported in a study conducted by Ravzanaadii, Kim, Choi, Hong and Kim [52]. The consumption of mealworms in developing countries could reduce the prevalence of iron and zinc deficiencies [53]. Iron deficiency is a major public health burden in African children [54]. Iron is an essential mineral for almost all living organisms, it participates in metabolic process; however, too much iron can lead to life threatening condition and tissue damage [55]. Anemia and iron overload are some of the common disorders that affect humans which can be related to low or high iron concentration in the body [55]. 

The amino acid profile of mealworms in this study agrees with previously published results [28]. The protein quality is based on amino acid profiles and digestibility [49]. When comparing the amino acid composition of different dried mealworm samples we found that the sun dried amino acid values were slightly higher than those of oven and freeze dried samples. This could have been influenced by the percentage of dry matter, Table 1 shows that the sun-dried samples dry matter was lower than freeze and oven dried samples. The findings of the current study show that the amino acid profile of mealworms is comparable to those of both soybean and fishmeal. Methionine was found to be the lowest amino acid in all dried mealworm samples; it is evident that insects are low in methionine [56]. Therefore, a commercial synthetic methionine should be added to poultry feed if mealworms will be used as a protein supplement. Moreover, the amino acid results are within the requirements of both human and animal daily recommended intake. The human nutrition daily recommended intake of amino acid is methionine 16.0 mg/g protein; threonine 23 mg/g protein and lysine 45.0 mg/g protein [57]. The threonine content for this study (2.77 g/100 g) was slightly higher compared with the previous investigation by Jajic et al. [51]. Amino acid is important in healing processes therefore, the deficiency of essential amino acid in food or feed may hinder healing recovery process [58,59]. It is therefore important to understand that essential amino acids cannot be manufactured in human bodies but can be obtained in food such as mealworm meal and others [59]. The content of tryptophan was not determined in this study. 

The dried mealworm fatty acid profile noted in this study is coherent with those obtained in other studies [18]. Barroso et al. [60] reported that the stages of development and harvesting could affect greatly the lipid content of the insect. The different types of diets used for rearing mealworms can also be another influential factor. According to Zornig et al., [61] these essential fatty acids will be enough for broilers’ growth because they require linoleic acid at levels of less than 0.20% of the total diet. Based on the results of this study, it was found that dried mealworms are rich in mono and polyunsaturated fatty acids compared to saturated fatty acids. Similar results were reported by Finke [62]. The omega 6 fatty acids (*n*-6) which play a key role in management and prevention of diseases such as type 2 diabetes in humans [63] was found to be between the required ranges. 

## 5. Conclusions

Developing an affordable drying technique will assist in preserving the quality of the final product of yellow mealworms. This will contribute to increasing the shelf life of mealworms, as currently available technologies are expensive for resource-poor, insect-rearing farmers. It can thus be deduced from the current study that sun drying yields the same nutritional quality and composition of mealworms as the oven and freeze-drying methods. Further research on the impact of substrate on the microbiota of yellow mealworms, hygiene measures and other rearing practices is necessary in order to provide additional strategies for small-scale, mealworm-rearing farmers to ensure food safety and quality of their products. Moreover, sun drying of yellow mealworms is a method that needs to be further explored in particular against the international food safety standard. The participatory technology development model could be used to design and make a drying technology suitable for small-scale, insect-rearing farmers. The process of freezing yellow mealworms followed by 20 s of blanching have been reported to reduce microbial counts and inactivate degradative enzymes responsible for food poisoning and spoilage. However, blanching treatments of mealworms should be standardized to improve antimicrobial effects with minimal quality. Furthermore, the effect of mealworm color on consumer preference should be evaluated.

## Figures and Tables

**Table 1:** Nutrient composition (g/100 g) of dried mealworm larvae.

Parameter	Sun-Dried	Freeze-Dried	Oven-Dried	SEM
Dry matter	89.44 ^c^	91.05 ^b^	93.33 ^a^	0.030
Ash	4.15 ^a^	4.23 ^a^	4.15 ^a^	0.031
Crude Protein	50.96 ^b^	51.45 ^a^	51.51 ^a^	0.016
Crude Fibre	6.20 ^a^	6.04 ^c^	6.11 ^b^	0.013
Gross Energy	24.75 ^a^	24.00 ^a^	24.63 ^a^	0.012

Values are means of duplicate analyzed mealworm samples. ^a,b,c^ Means in the same column with different superscripts are significantly different (*p* < 0.05).

**Table 2:** Color values of oven-dried, sun-dried and freeze-dried mealworm larvae.

Parameter	Sun-Dried	Freeze-Dried	Oven-Dried	SEM
L* (lightness)	30.95 ^c^	36.18 ^b^	42.02 ^a^	1.007
a* (Redness)	2.32 ^a^	4.24 ^a^	4.75 ^a^	0.924
b* (yellowness)	−1.47 ^c^	6.54 ^a,b^	12.35 ^a^	1.507

Values are means of duplicate analyzed mealworm samples. ^a,b,c^ Means in the same column with different superscripts are significantly different (*p* < 0.05).

**Table 3:** Mineral composition (mg/kg) of dried mealworm larvae.

Parameter	Sun-Dried	Freeze-Dried	Oven-Dried	SEM
Calcium	275.01 ^c^	282.45 ^b^	294.77 ^a^	0.015
Magnesium	2220.10 ^b^	2174.76 ^c^	2458.60 ^a^	0.059
Copper	16.00 ^b^	16.00 ^b^	17.47 ^a^	0.008
Iron	50.00 ^b^	66.97 ^a^	46.45 ^c^	0.012
Manganese	11.75 ^a^	11.55 ^a,b^	11.07 ^b^	0.122
Zinc	121.49 ^a^	118.97 ^c^	121.41 ^b^	0.013
Potassium	8201.00	8149.00	7244.00	779.431
Sodium	1080.12 ^b^	964.90 ^c^	1089.22 ^a^	0.012
Phosphorus	6899.82 ^b^	6712.90 ^c^	7484.15 ^a^	0.087

Values are means of duplicate analyzed mealworm samples. ^a,b,c^ Means in the same column with different superscripts are significantly different (*p* < 0.05).

**Table 4:** Amino acid values of dried mealworm larvae (g/100 g).

Parameter	Sun-Dried	Freeze-Dried	Oven-Dried	SEM
Histidine	1.60 ^a^	1.60 ^a^	1.27 ^b^	0.009
Arginine	3.17 ^a^	2.71 ^b^	2.60 ^c^	0.010
Serine	2.80 ^a^	2.01 ^c^	2.53 ^b^	0.010
Glycine	2.91 ^a^	2.55 ^b^	2.91 ^a^	0.013
Aspartic	5.72 ^a^	3.57 ^c^	4.87 ^b^	0.015
Glutamine	8.56 ^a^	5.54 ^c^	7.01 ^b^	0.013
Threonine	2.77 ^a^	1.90 ^c^	2.56 ^b^	0.012
Alanine	4.23 ^a^	3.20 ^b^	4.20 ^a^	0.008
Proline	5.20 ^b^	4.91 ^c^	5.36 ^a^	0.012
Lysine	4.40 ^a^	2.55 ^c^	3.95 ^b^	0.012
Tyrosine	4.33 ^c^	4.91 ^a^	4.80 ^b^	0.010
Methionine	0.76 ^a^	0.60 ^b^	0.80 ^a^	0.009
Valine	3.65 ^a^	3.00 ^c^	3.40 ^b^	0.009
Isoleucine	2.51 ^a^	2.13 ^c^	2.44 ^b^	0.013
Leucine	3.84 ^b^	3.56 ^c^	3.60 ^a^	0.015
Phenylalanine	3.46 ^b^	2.95 ^c^	3.57 ^a^	0.015

Values are means of duplicate analyzed mealworm samples. ^a,b,c^ Means in the same column with different superscripts are significantly different (*p* < 0.05).

**Table 5:** Fatty acids profile of oven-dried, sun-dried and freeze-dried mealworm larvae.

Parameter	Sun-Dried	Oven-Dried	Freeze-Dried	SEM
% Fat	27.26 ^a^	25.73 ^c^	26.23 ^b^	0.015
% FFDM	69.31 ^a^	67.75 ^b^	51.75 ^c^	0.122
% Moisture	3.37 ^c^	6.33 ^b^	21.82 ^a^	0.015
FAME (% of total fatty acids):				
Lauric (C12:0)	0.14	0.11	0.13	0.012
Tridecoic (C13:0)	0.01 ^b^	0.00 ^c^	0.02 ^a^	0.000
Myristic (C14:0)	2.21 ^a^	2.01 ^a^	2.16 ^b^	0.012
Pentadecylic (C15:0)	0.13	0.12	0.14	0.150
Palmitic (C16:0)	17.6 ^a^	17.615 ^a^	17.32 ^a^	0.087
Palmitoleic (C16:1c9)	1.23 ^a^	1.15 ^a^	1.11 ^a^	0.087
Margaric (C17:0)	0.16 ^a,b^	0.13 ^b^	0.20 ^a^	0.012
Stearic acid (C18:0)	2.94 ^b^	3.01 ^a^	3.05 ^a^	0.012
Oleic (C18:1C9)	36.14 ^b^	36.75 ^a^	35.5 ^c^	0.12
Nonoadecanoic (C19:0)	0.02	0.03	0.02	0.012
Linolelaidic (C18:2t9, 12 (*n*-6)	0.02	0.03	0.03	0.012
Linoleic (C18:2c9,12 (*n*-6)	37.21 ^b^	36.75 ^c^	38.44 ^a^	0.013
Arachidic (C20:0)	0.06	0.07	0.06	0.015
α-Linolenic (C18:3c9,12,15(*n*-3)	1.54 ^b^	1.67 ^a^	1.52 ^b^	0.013
Eicosadienoic (C20:2c11,14 (*n*-6)	0.02	0.02	0.02	0.000
Eicosatrienoic (C20:3c8,11,14 (*n*-6)	0.01 ^b^	0.02 ^a^	0.01 ^b^	0.000
Eicosatrienoic (C20:3c11, 14,17 (*n*-3)	0.02 ^b^	0.03 ^a^	0.02 ^b^	0.000
Arachidonic (C20:4c5,8,11,14 (*n*-6)	0.00 ^b^	0.01 ^a^	0.01 ^a^	0.000
Nervonic (C24:1c15)	0.01	0.01	0.01	0.000
Total Saturated Fatty Acids (SFA)	23.45	23.10	23.22	0.104
Total Mono Unsaturated Fatty Acids (MUFA)	37.54 ^b^	38.21 ^a^	36.62 ^c^	0.013
Total Poly Unsaturated Fatty Acids (PUFA)	38.83 ^a,b^	37.71 ^b^	40.11 ^a^	0.502
Total Omega- 6 Fatty Acids (*n*-6)	37.25	37.71	38.54	0.485
Total Omega-3 Fatty Acids (*n*-3)	1.56 ^b^	1.71 ^a^	1.54 ^b^	0.01
PUFA: SFA	1.63 ^b^	1.64 ^b^	1.71 ^a^	0.015
*n*-6/*n*-3	23.64 ^b^	21.55 ^c^	24.67 ^a^	0.015

Values are means of duplicate analyzed mealworm samples. ^a,b,c^ Means in the same column with different superscripts are significantly different (*p* < 0.05).

## Data Availability

Data is contained within the article.
